# Subsurface plumbing system architecture in the South Makassar Basin, offshore Indonesia, and its implications for methane emissions and geological storage

**DOI:** 10.1038/s41598-026-39597-y

**Published:** 2026-02-16

**Authors:** Harya D. Nugraha, S. N. Fathiyah Jamaludin, Ryo Matsumoto, Shiro Ohkawa, Hitoshi Tomaru, Imam Juanda, Ida Herawati, Weny Astuti, Syahreza S. Angkasa

**Affiliations:** 1 Center for Sustainable Geoscience and Outreach (CSGO), Universitas Pertamina, Jakarta, Indonesia; 2 Department of Earth Science, University of Bergen, Bergen, Norway; 3 Petroleum Engineering Department, Universiti Teknologi PETRONAS, Seri Iskandar, Malaysia; 4 Gas Hydrate Research Laboratory (GHRL), Meiji University, Tokyo, Japan; 5 Department of Earth Sciences, Chiba University, Chiba, Japan; 6 Faculty of Exploration and Production Technology, Universitas Pertamina, Jakarta, Indonesia; 7 Research Center for Geological Resources, National Research and Innovation Agency, Jakarta, Indonesia

**Keywords:** Energy science and technology, Engineering, Solid Earth sciences

## Abstract

Subsurface plumbing systems in deep-marine basins influence gas hydrate distribution, natural methane emissions, and seal integrity relevant to subsurface CO₂ storage. Using high-quality, post-stack time-migrated (PSTM) 3D seismic reflection and well data from the South Makassar Basin, offshore Indonesia, we characterised both focused and unfocused fluid-flow features within a fine-grained seal succession that form endmembers of a fluid-flow continuum. A focused system is expressed by fluid pipes rooted at carbonate structural highs and, in many cases, terminating at the seabed as pockmarks. An unfocused system is expressed by laterally extensive polygonal and radial fault networks that spatially coincide with laterally continuous bottom-simulating reflections (BSRs), indicating gas hydrate occurrence. Based on mainly seismic reflection data, we propose a four-stage evolutionary model for focused flow, from radial faulting centred at structural highs, followed by pipe formation, and eventual seal breaching expressed as pockmarks on the seabed. These stages define a structurally mediated continuum of seal bypass development that is probably modulated by reservoir pressure buildup, reservoir geometry, and mechanical heterogeneity of the overburden. These findings provide new insights into the architecture of subsurface plumbing systems in the South Makassar Basin and has implications for natural methane seepage and long-term subsurface storage there and elsewhere.

## Introduction

Subsurface fluid flow is ubiquitously expressed in deep-marine basins. It influences a range of critical processes, including hydrocarbon accumulation, gas hydrate formation, natural methane seepage, and the long-term security of engineered subsurface storage (e.g., carbon capture and storage or CCS)^1–3^. In addition to supporting resource accumulations, fluid migration pathways are directly linked to climate-relevant outcomes. Methane, a potent greenhouse gas, can be released through natural seepage or hydrate destabilisation in response to ocean warming^4,5^. The failure of sealing intervals may also compromise CCS integrity^3^, allowing CO₂ leakage that undermines mitigation efforts^6,7^. These risks highlight the importance of constraining fluid migration mechanisms through sealing units in sedimentary basins^3,8,9^. Therefore, understanding the architecture of fluid migration pathways is central to both energy exploration and climate mitigation strategies.

Although fine-grained marine sediments are often assumed to act as effective regional seals due to their low permeability and high sealing capacity, they frequently host complex fluid migration systems^1,3,10–14^. Subsurface fluid migration may follow focused (e.g., Berndt^2^) or unfocused pathways (e.g., Cartwright, et al. ^15^). Focused flow occurs through chimney-like conduits (e.g., fluid pipes) that cut across stratigraphy (e.g., Cartwright and Santamarina^16^), channelling methane-rich fluids efficiently to the seafloor, forming pockmarks and potentially leading to localised hydrate accumulation and subsequent dissociation^17–20^. In contrast, unfocused fluid migration is mediated by pervasive polygonal fault systems that may act as both conduits and baffles, modifying or delaying vertical fluid movement^15,21,22^. Investigating both spatial and temporal attributes of these features is essential to understanding how fluid plumbing systems evolve. Present-day seabed features provide insights into active fluid systems (e.g., Hovland, et al. ^23^). Although their long-term behaviour remains uncertain, such features are observed in many deep marine areas such as the Gulf of Mexico^18^, offshore Nigeria^11^, offshore Namibia^24^, offshore New Zealand^25,26^, offshore Norway^27^, offshore Angola^28^, offshore Japan^29,30^, and South China Sea^31^. Despite these extensive examples, many of these active plumbing systems remain poorly resolved, especially in basins where only 2D seismic data are available.

**Fig. 1:**
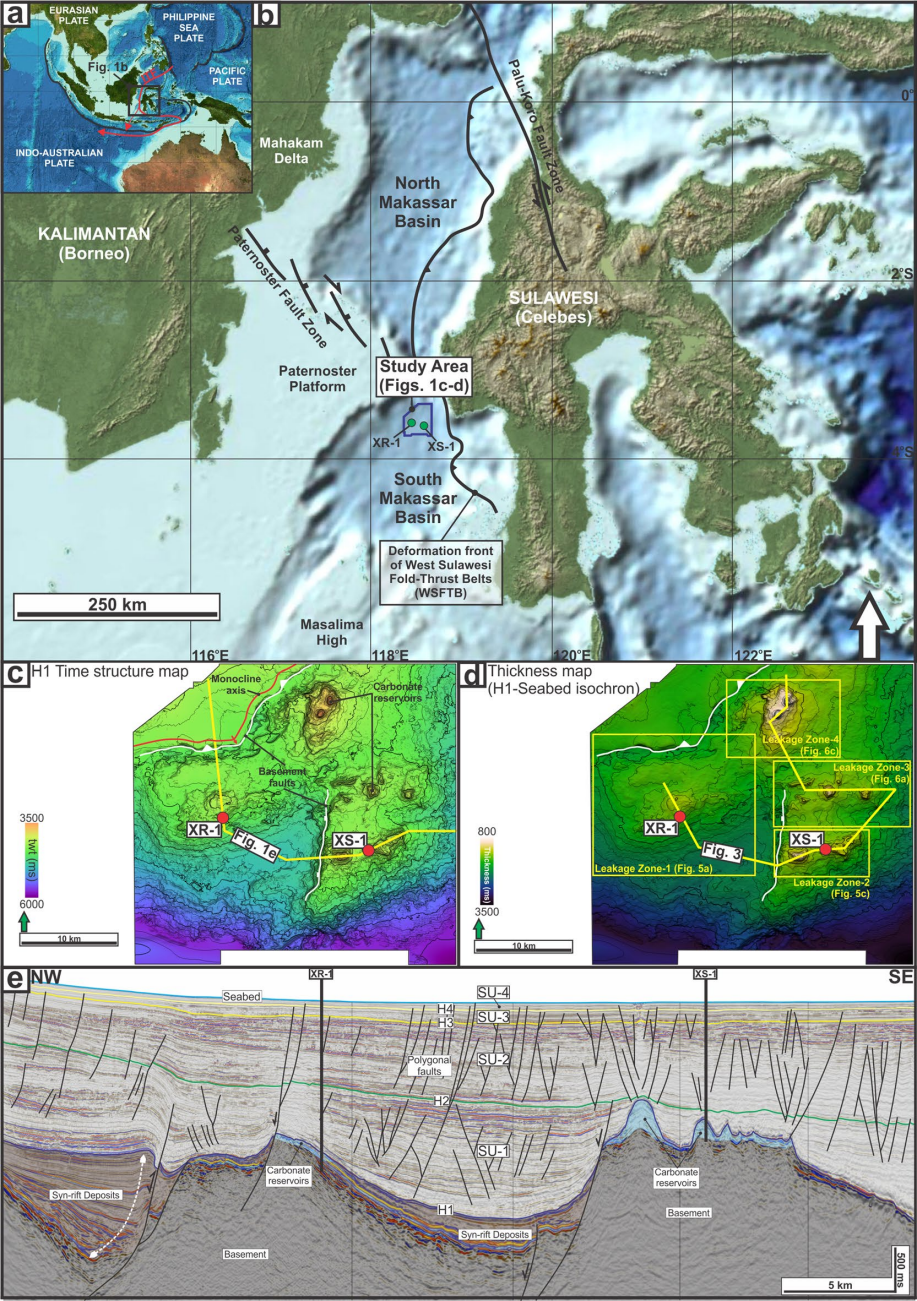
Geological setting of the study area. (**a**) Regional map showing the Makassar Strait in a tectonically active setting and its role as a main pathway for the Indonesian Throughflow (ITF). (**b**) Location of the study area. The dark-blue polygon outlines the 3D seismic survey, and green circles mark the two wells. Key adjacent structures include the Paternoster Fault Zone to the west and the West Sulawesi Fold–Thrust Belt to the east^41,43,95^. (**c**) Two-way travel time (TWT) structure map of horizon H1, highlighting the structure of carbonate reservoirs. (**d**) Thickness (isochron) map between H1 and the seabed, illustrating thickness variations of the study interval. (**e**) Interpreted seismic section showing the geometry of the study interval (i.e., post-rift succession) and the underlying syn-rift and basement units.

The South Makassar Basin, offshore Indonesia, offers a natural laboratory for studying the subsurface plumbing systems and their surface expressions (Fig. 1a-b). Pockmarks and bottom-simulating reflections (BSRs) identified from seismic reflection data^32^, seep geochemistry from drop-core samples consistent with a thermogenic petroleum system^33,34^, and hydrate samples recovered from shallow piston cores adjacent to the pockmarks^35,36^suggest active fluid expulsion from deep carbonate reservoirs^33^. These fluids migrate through over 1000 m of fine-grained marine sediment, breaching even low-permeability mass-transport deposits (MTDs) commonly assumed to enhance sealing^37–39^. Previous studies in the basin have identified hydrate play types that amount to the potential reserve of 105 trillion cubic feet (TCF) of methane resources^32^and constrained its thermal conditions^40^. However, the spatial relationship between deep reservoirs and seabed expressions remains poorly understood as the earlier works relied only on 2D seismic reflection data, which limit the ability to delineate the detailed geometry and maturity of fluid migration pathways.

In this study, we use high-quality, post-stacked time-migrated (PSTM) 3D seismic reflection data integrated with two exploration wells to investigate the architecture of the subsurface plumbing system in the South Makassar Basin. The objectives are: (1) to provide the stratigraphic and structural framework of the sealing interval; (2) to document and interpret vertically focused and laterally extensive fluid flow features; and (3) to discuss the implications of these features for natural methane emissions and long-term storage security. These findings improve our understanding of how active plumbing systems operate in deepwater fine-grained basins and support future exploration and monitoring efforts for both energy resources and climate mitigation.

## Geological setting

The South Makassar Basin is part of an extensional system located in the southern Makassar Strait, a 200 to 2000 m deep marine corridor separating the islands of Borneo and Sulawesi (Fig. 1). It is situated in a tectonically active region, where Eurasian, Pacific, Philippine Sea plates interact (Fig. 1a). It is bounded to the north by a narrow channel connecting it to the North Makassar Basin, to the south by the Masalima High, to the east by the West Sulawesi Fold-Thrust Belt (WSFTB), and to the west by the Paternoster Platform^41–43^(Fig. 1b). A southward-flowing current that carries water from the Pacific to the Indian Ocean, i.e., known as the Indonesian Throughflow (ITF), crosses the strait at a relatively high velocity of 1 m/s^44^ (Fig. 1a).

### Tectonic evolution

The basin’s tectonic history was preconditioned by Cretaceous compressional events, which established structural fabrics later exploited by the subsequent rifting process^45^. The syn-rift phase began in the Eocene^46^, resulting in the formation of horsts and grabens infilled with coarse-grained and organic-rich fine-grained clastics^33,35,47^. Carbonate platforms and build-ups developed atop structural highs, contemporaneous with hemipelagic sedimentation in adjacent areas, marking the transgressive onset of the post-rift phase^35,47^(Fig. 1c-e). Since the Late Oligocene, post-rift thermal subsidence has led to the predominant deposition of deep-marine claystones, continuing to the present day^33,35,47^. A major compressional phase associated with the WSFTB began during the Miocene^47,48^(Fig. 1b).

### Petroleum systems

The basin hosts a proven petroleum system. It is characterised by organic-rich fine-grained sediments deposited during the syn-rift phase as the primary source rocks, while carbonate build-ups on structural highs serve as the main reservoirs^33,35,47,49^(Fig. 1e). These reservoirs are sealed by over 1 km of deep-marine claystone succession^33,35^(Fig. 1d,e). The generation of petroleum is interpreted to be controlled by tectonic loading of the WSFTB and thus a critical moment in the Late Pliocene^47^. The gas migrated towards carbonate reservoirs, one of which was drilled at XS-1 (Fig. 1c-e), where 102 m-thick of gas column (97% methane) were discovered^33,47^. On the other hand, faulted carbonate platform reservoirs penetrated by XR-1 were dry^33,47^(Fig. 1c-e). The gas origin is interpreted as predominantly thermogenic based on stable isotope analysis^33,34^with a possible mixing with biogenic gas^47,50^. Vertical fluid migration through the seal and overburden has breached the seabed, as evidenced by a shallow geohazards survey^36^.

### Gas hydrates

Gas hydrates have been documented in the basin through both physical sampling and remote detection. Recovered hydrate samples were found alongside chemosynthetic clam communities, indicating active fluid expulsion^35,36^. Bottom Simulating Reflections (BSRs) mapping by Dirgantara, et al. ^32^ identified hydrate accumulations associated with slope, polygonal faults, sediment waves, buried carbonates, and fold structures, with an estimated methane resource of 105 TCF. Treating BSRs as the base of the gas hydrate stability zone (BGHSZ) and assuming 1D, steady-state conductive heat flow (linear geothermal gradient), Dirgantara, et al. ^40^ used methane-hydrate phase equilibrium (constant seawater salinity, constant gas composition, hydrostatic pore pressure) to estimate a mean heat flow of 86 mW/m², a geothermal gradient of 73 °C/km, and an average thermal conductivity of 1.19 W/m·°C across water depths of 400–2050 m. These parameters place the theoretical BGHSZ depth at c.218 m below the seafloor. They note a 6–19 m discrepancy between the estimated BGHSZ depth and the values reported in well reports, attributed to uncertainties in methodology, theoretical assumptions, and local effects such as thermal uplift and fluid advection^40^. Despite evidence of gas accumulations in the carbonate reservoirs, gas hydrate occurrence, and associated leakage to the seabed, the architecture of the plumbing systems connecting these features remains poorly understood; an issue this study aims to address.

## Data and methods

### Data

This study is based on high-quality, post-stack time-migrated (PSTM) 3D seismic and well data (Fig. 1b). The seismic volume covers 990 km², has a bin spacing of 25 m × 12.5 m (inline × crossline), and a 4 ms sample interval. The processed time window spans 1.8–6 s two-way travel time (TWT), corresponding to 1026 samples per trace; however, this study focuses on the interval shallower than 4.9 s TWT. The 3D seismic data are zero-phase with Society of Exploration Geophysicists (SEG) normal polarity, where increases in acoustic impedance are expressed as positive amplitudes (red). Well-derived water velocity is 1492 m/s, and near-seabed sediments (< 200 ms below the seafloor) have a dominant frequency of 50 Hz, yielding a vertical resolution of c.7.5 m. The study interval from the seafloor to the top of the carbonate reservoirs has an average velocity of 1829 m/s, with lower dominant frequency of 30 Hz, resulting in a lower vertical resolution of c.15 m. The velocity data are from zero-offset, rig-source vertical seismic profiling (VSP) survey conducted at the wells.

**Fig. 2:**
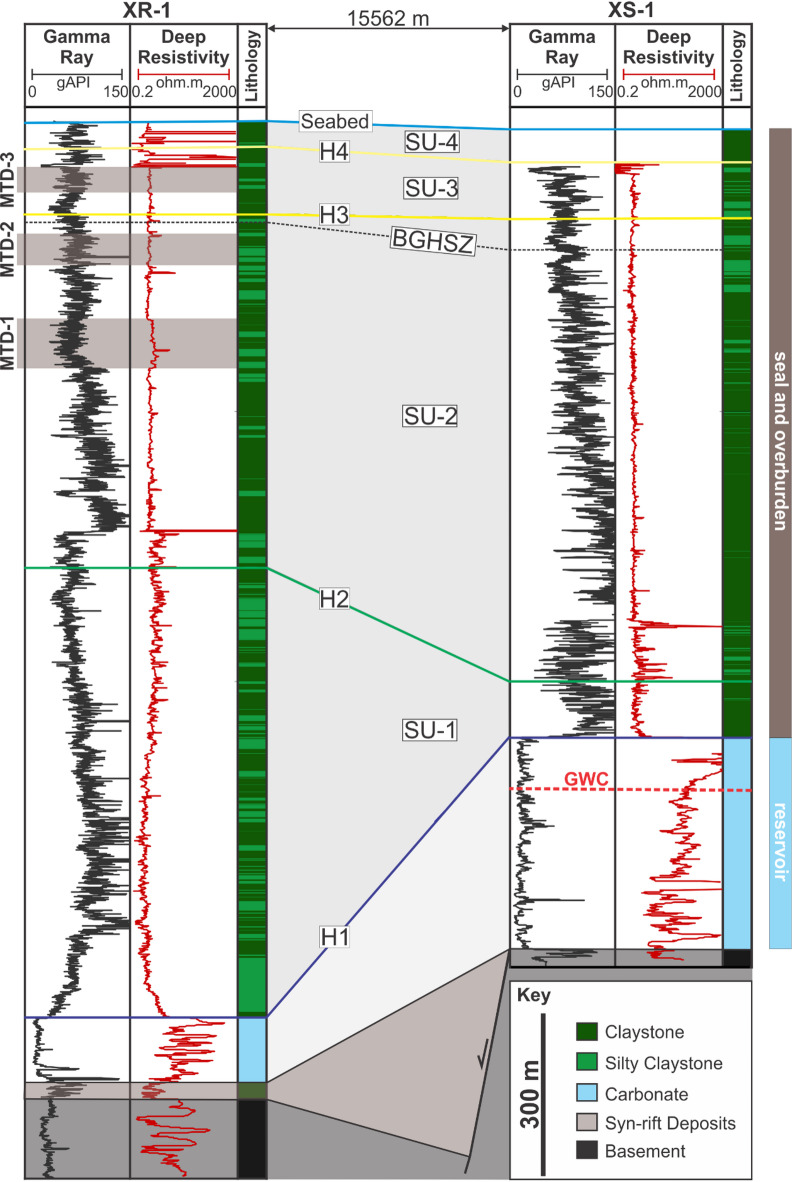
Well log correlation between XR-1 and XS-1. MTDs: Mass-transport deposits; BGHSZ: Base of Gas Hydrate Stability Zone; SU: Seismic Unit; H: Horizon; GWC: Gas-Water Contact.

Two exploration wells (XR-1 and XS-1) provide gamma ray and deep resistivity logs that serve as stratigraphic controls for interpreting the studied interval (Fig. 2). Additionally, biostratigraphic data from cuttings, i.e., only recovered from 881 m to 204 m above the top carbonate at XR-1 and XS-1, respectively, constrain the age and depositional water depth of the interval.

### Methods

#### Seismic interpretation and attributes analysis

**Fig. 3:**
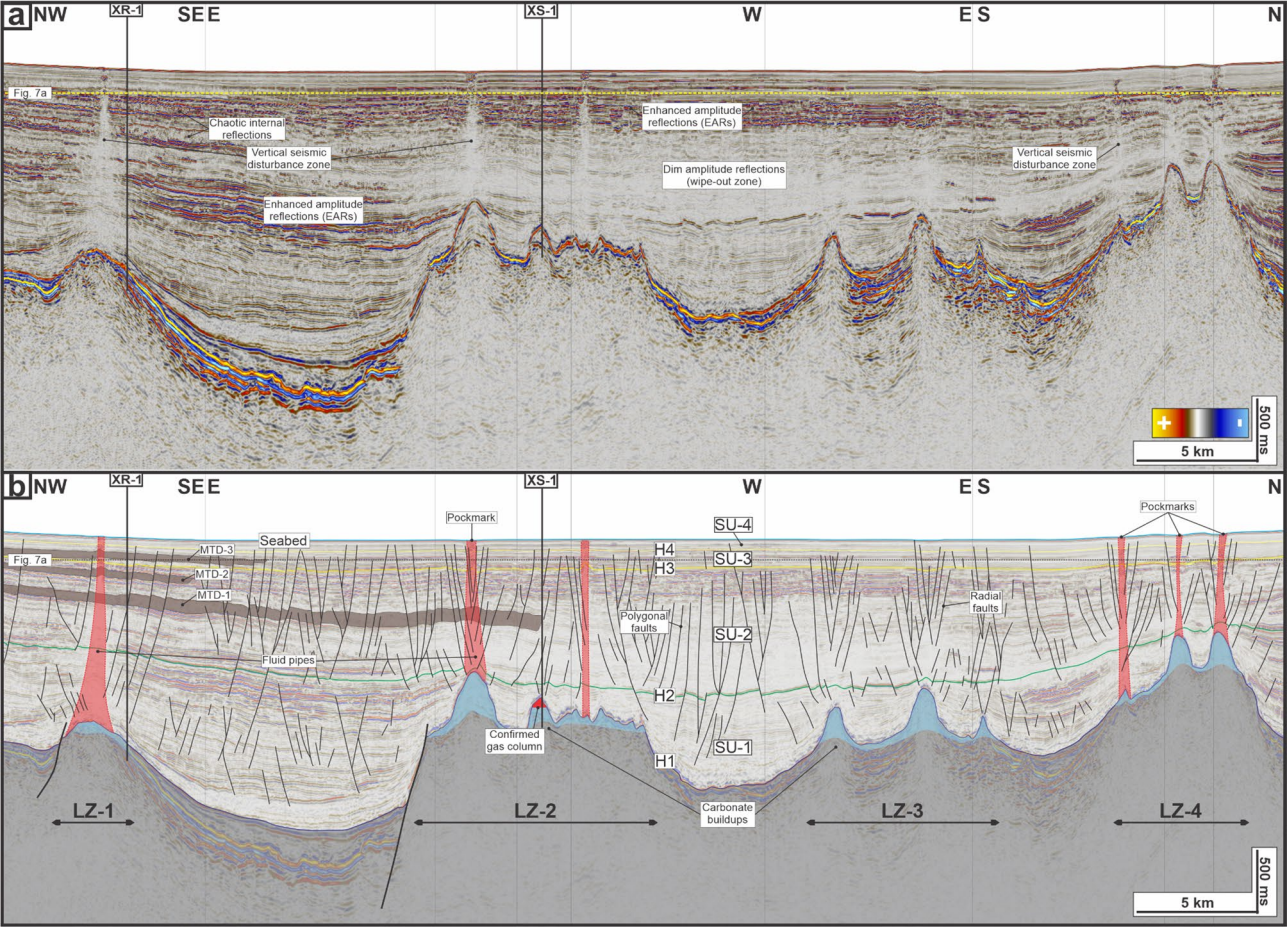
(**a**) Uninterpreted and (**b**) interpreted seismic sections depicting an overview of leakage zones (LZs) and associated fluid flow features in the study area.

Seismic interpretation was carried out on the 3D seismic reflection volume to establish a stratigraphic and structural framework of the study area (Fig. 3). Four major seismic units (SU-1 to SU-4) defined by five horizons (H1-H4 and the seabed) were interpreted based on reflection terminations, bounding surface characters, and internal architecture^51^. These interpretations were tied to well by the checkshot data and constrained by biostratigraphic markers and gamma-ray and deep resistivity log responses (Fig. 2).

Two seismic attributes were extracted to assist the stratigraphic and structural interpretation. Variance attributes were applied to highlight discontinuities, so that fault patterns can be clearly observed^52^. Spectral decomposition with red-green-blue (RGB) blending was used to enhance structural discontinuities and amplitude anomalies by combining three band-limited frequency volumes displayed as red (low), green (mid), and blue (high) components^53,54^. The frequencies of 36, 44, and 53 Hz were selected from the data’s amplitude spectrum to represent low-, mid-, and high-frequency bands, respectively. As seismic expression is frequency dependent, separating bands can emphasise features of different characteristic scale and tuning: higher frequencies tend to sharpen small-scale edges and discontinuities (e.g., minor faults), whereas lower frequencies more readily highlight broader responses and may be sensitive to attenuation (e.g., gas-charged intervals). Colour intensity reflects the relative spectral power within each band; red hues indicate low-frequency dominance, while features with strong responses across all three bands appear white due to comparable contributions from red, green, and blue^55^. In this study, RGB blends are therefore used as a qualitative aid to map fault-related discontinuities and visualise amplitude heterogeneity around fluid flow features. 

#### Quantitative analysis

Quantitative morphometric analysis was conducted to characterise the key fluid-flow features following established approaches13. Vertical disturbance zone (fluid pipe) vertical extent (height) was measured in two-way travel time and converted to metres using the average interval velocity (1829 m/s) for the seafloor-H1 interval. Pipe root width was measured in map view from horizon slices/attribute maps. Seabed depression (pockmark) diameter was measured in map view, and depth was calculated from relief in two-way travel time converted using water velocity (1492 m/s). Given the limitations of the time-domain nature of the PSTM 3D seismic dataset and the vertical resolution (c. 7.5–15 m), the vertical morphometric measurements are treated as first-order depth estimates. To investigate the orientation of small-offset faults, we generated variance attributes to highlight small-offset fault-related discontinuities at selected horizons25,56,57. Small-offset fault-related lineaments were then extracted from the variance maps and rose diagrams were generated from the resulting azimuths.

## Results

### Major structural elements

The structural configuration of the study area is dominated by two basement-related faults (Fig. 1c). In the northwest, a normal fault reactivated as a thrust (i.e., an inverted fault) trends E-W (090–270°) in its western segment and changes to a NE-SW trend (045–225°) toward the east. This fault bounds a prominent monocline at H1 (the base of the study interval; Fig. 1c-e). In the central part of the study area, a N-S–trending (000–180°) fault separates two basement highs. The isochron map between H1 and the seabed shows marked variations in post-rift thickness over structural highs (c. 800–1500 ms or c.732–1372 m) and fault-bounded depocenters (> 1900 ms or > 1738 m; Fig. 1d). These basement structures controlled the location of carbonate reservoirs, which are overlain by the post-rift intervals (Fig. 1e). Spatial clusters of the carbonate reservoirs define four focus areas (hereafter leakage zones, LZ-1 to LZ-4; Figs. 1d and 3).

### Seismic stratigraphic framework

Five mapped seismic horizons (H1-H4 and the seabed) define four regionally mappable seismic units (SU-1 to SU-4) within the post-rift succession (Figs. 1e and 3). H1 marks the top of the Late Oligocene carbonate reservoirs and the base of the post-rift interval (Fig. 3), and therefore forms the base of SU-1 (H1-H2). SU-1 is characterised by subparallel reflections that are locally faulted, with generally dim amplitudes over basement highs (Figs. 1e and 3b), and it thins above basement highs and thickens into adjacent depocentres (Fig. 1d-e). At the base of SU-1, well-log responses indicate a lithological shift from carbonates below (low gamma-ray, and high resistivity) to more clay-rich sediments above (serrated, predominantly high gamma-ray, and low resistivity) (Fig. 2).

H2 is a prominent intra-post-rift surface mapped as the top of the main sealing interval in the study area (Figs. 1e and 3b) and forms the base of SU-2 (i.e., bounded by H2 and H3). SU-2 thickens eastward away from the H1 monocline (Fig. 1e). Within SU-2, seismic sections image internally disrupted, chaotic-to-transparent packages with irregular top and base surfaces, contrasting with the surrounding sub-parallel reflections (Fig. 3). These packages are interpreted as mass-transport deposits (i.e., MTD-1 and MTD-2) and are penetrated by XR-1 (Fig. 2) but are not observed at XS-1 (Fig. 3b). At XR-1, MTD-1 is 87 m thick and exhibits lower gamma-ray and elevated resistivity, whereas MTD-2 is 51 m thick and shows a more subtle gamma-ray and resistivity anomaly (Fig. 2).

**Fig. 4:**
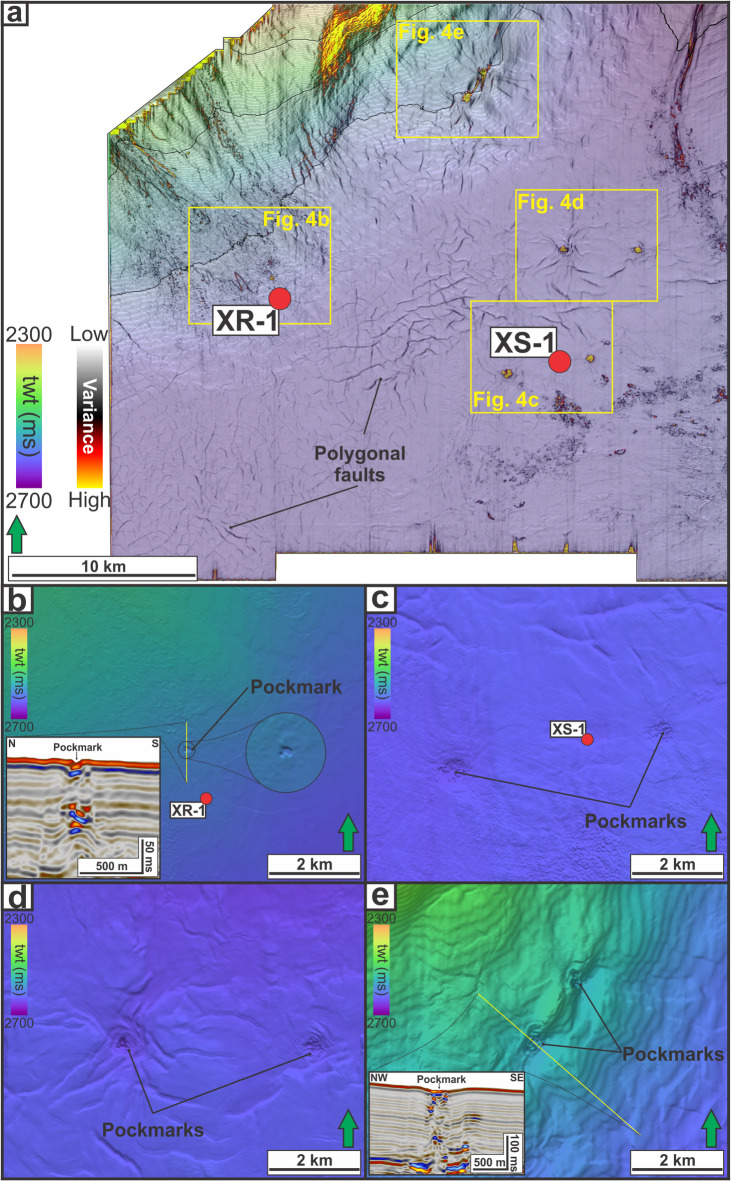
(**a**) Time structure map of seabed overlain by a variance attribute, highlighting seafloor lineaments (e.g., polygonal faults) and circular seabed depressions (interpreted as pockmarks). A close-up of seabed time structure map showing pockmarks within LZ-1 (**b**), LZ-2 (**c**), LZ-3 (**d**), and LZ-4 (**e**).

H3 and H4 are intra-Quaternary surfaces based on well reports and are distinguished by reflection character and by the degree of disruption by small-offset faults, with greater disruption at H3 than at H4 (Figs. 1e and 3b). These horizons define SU-3 (H3-H4) and SU-4 (H4-seabed), respectively. SU-3 and SU-4 show comparable eastward thickening in the illustrated sections (Fig. 1e), but SU-3 is more disrupted by small-offset faulting than SU-4 and hosts an additional mass-transport deposit (MTD-3), which is 46 m thick at XR-1 (Figs. 2 and 3b). The seabed horizon caps SU-4 and captures present-day bathymetry and shallow deformation features (Figs. 3b and 4a).

### Observations of fluid flow features

#### Seabed circular depressions (pockmarks)

Circular to sub-circular depressions on the seabed are observed across the study area (Fig. 4). The depressions occur within the four spatial clusters (i.e., LZ-1 to LZ-4, see Fig. 1d) and vary in diameter and depth, with measured diameters of 230–580 m and depths of 10–18 m. In LZ-1, there is a relatively small depression, i.e., 230 m in diameter and 11 m deep (Fig. 4b). In LZ-2, the depressions include the widest mapped examples: one in the west (580 m diameter, 10 m deep) and one in the east (450 m diameter and 12 m deep), of XS-1 (Fig. 4c). In LZ-3, the depressions are 345–450 m in diameter and 12–13 m deep (Fig. 4d). In LZ-4, the depressions are 362–556 m in diameter and are the deepest examples, i.e., 17–18 m (Fig. 4e). Hereafter, these seabed depressions are interpreted as pockmarks following their diagnostic seabed morphology^23,58^.

#### Vertical seismic disturbance zones (fluid pipes)

Vertical seismic disturbance zones are characterized by vertically extensive zone of reduced reflections continuity, with locally chaotic to transparent internal character relative to adjacent, more laterally continuous reflections. These zones can be observed in all four spatial clusters (i.e., LZ-1 to LZ-4; Fig. 3b). Based on these characters, these vertical seismic disturbance zones are termed fluid pipes^1,13,16^. For consistency, each pipe is described by its root (base), terminus (top boundary), and body (interval between root and terminus).

**Fig. 5:**
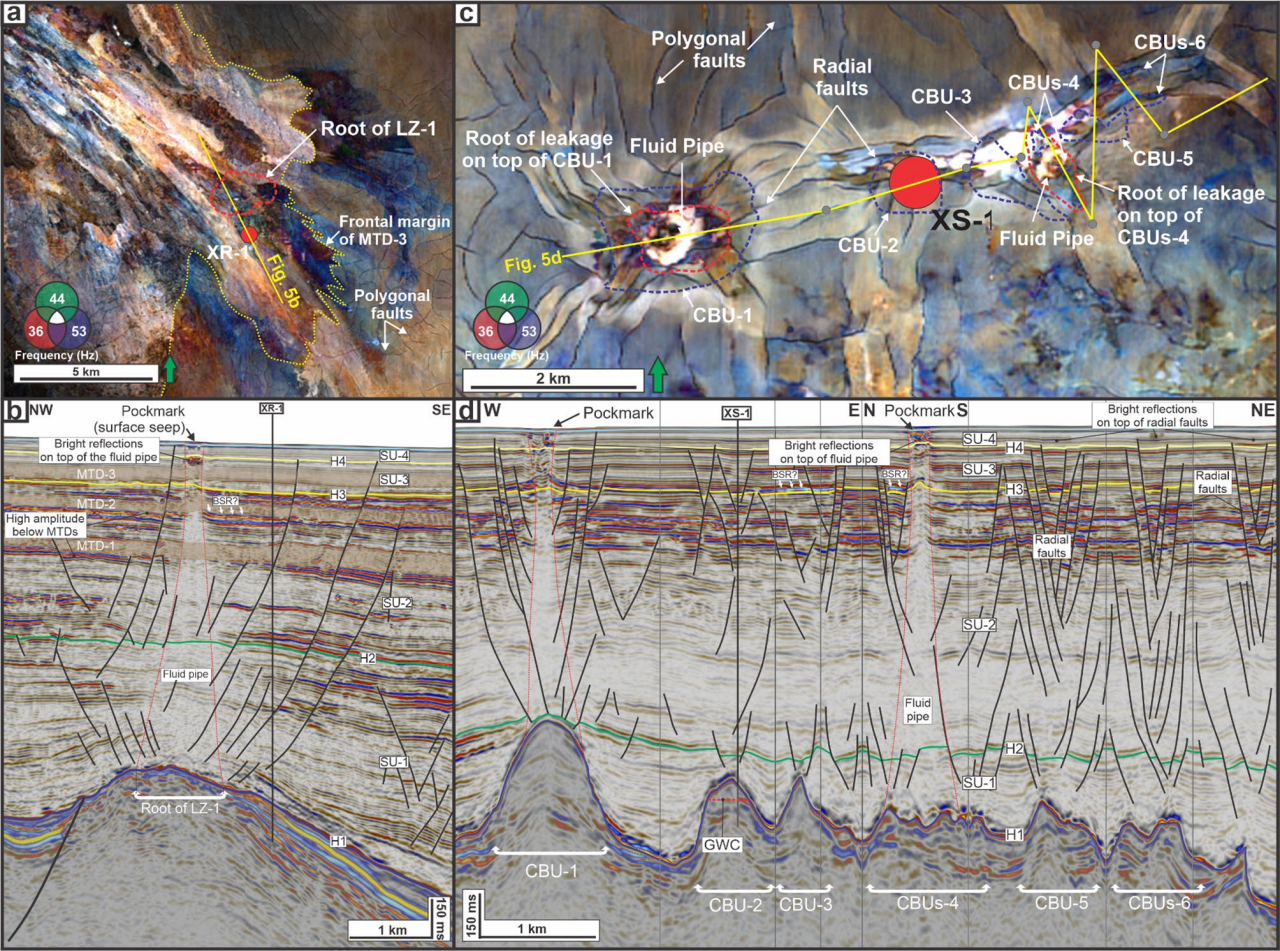
(**a**) LZ-1: RGB-blended spectral decomposition map extracted at horizon H3, showing vertical disturbance zones (interpreted as fluid pipes) that cut through MTD-3 and adjacent polygonal-fault lineaments. (**b**) Interpreted seismic section across LZ-1. (**c**) LZ-2: RGB-blended spectral decomposition map extracted at horizon H3, showing fluid pipes, small-offset faults (radial and polygonal), and the outlines of carbonate build-ups (CBUs). Only CBU-1 and CBU-4 spatially coincide with fluid pipes. (**d**) Interpreted seismic section across LZ-2.

Across the study area, the pipes extend upward from H1 toward the shallow section and in many cases intersect the seabed, with vertical extents of 899–1691 m and root widths of 496–1285 m. Pipe geometry ranges from relatively straight vertical forms to zones that narrow upward (Fig. 3b). In LZ-1, a pipe is first expressed at H1 above the faulted carbonate platform penetrated by XR-1 (Fig. 5a-b). The pipe extends upward for 1691 m and has a root width of 1285 m (Fig. 5a-b). The pipe intersects stacked MTD packages (Fig. 5b). The upper part of the pipe is characterized by the presence of bright reflections and coincides with a pockmark on the seabed.

In LZ-2, two pipes occur above carbonate build-up (CBU) crests (i.e., CBU-1 and CBUs-4; Fig. 5c-d). The pipe above CBU-1 extends upward for 1165 m with a root width of 823 m, whereas the pipe above CBUs-4 reaches 1584 m in height with a root width of 1209 m (Fig. 5c,d). Similar to the pipe of LZ-1, the upper part of these pipes shows bright reflections and alignment with pockmarks on the seabed (Fig. 5d). Other carbonate build-ups in this area do not show comparable fluid pipes, but radial-fault patterns with bright reflections on top of the CBUs are observed in both plan-view (Fig. 5c) and seismic section (Fig. 5d).

**Fig. 6:**
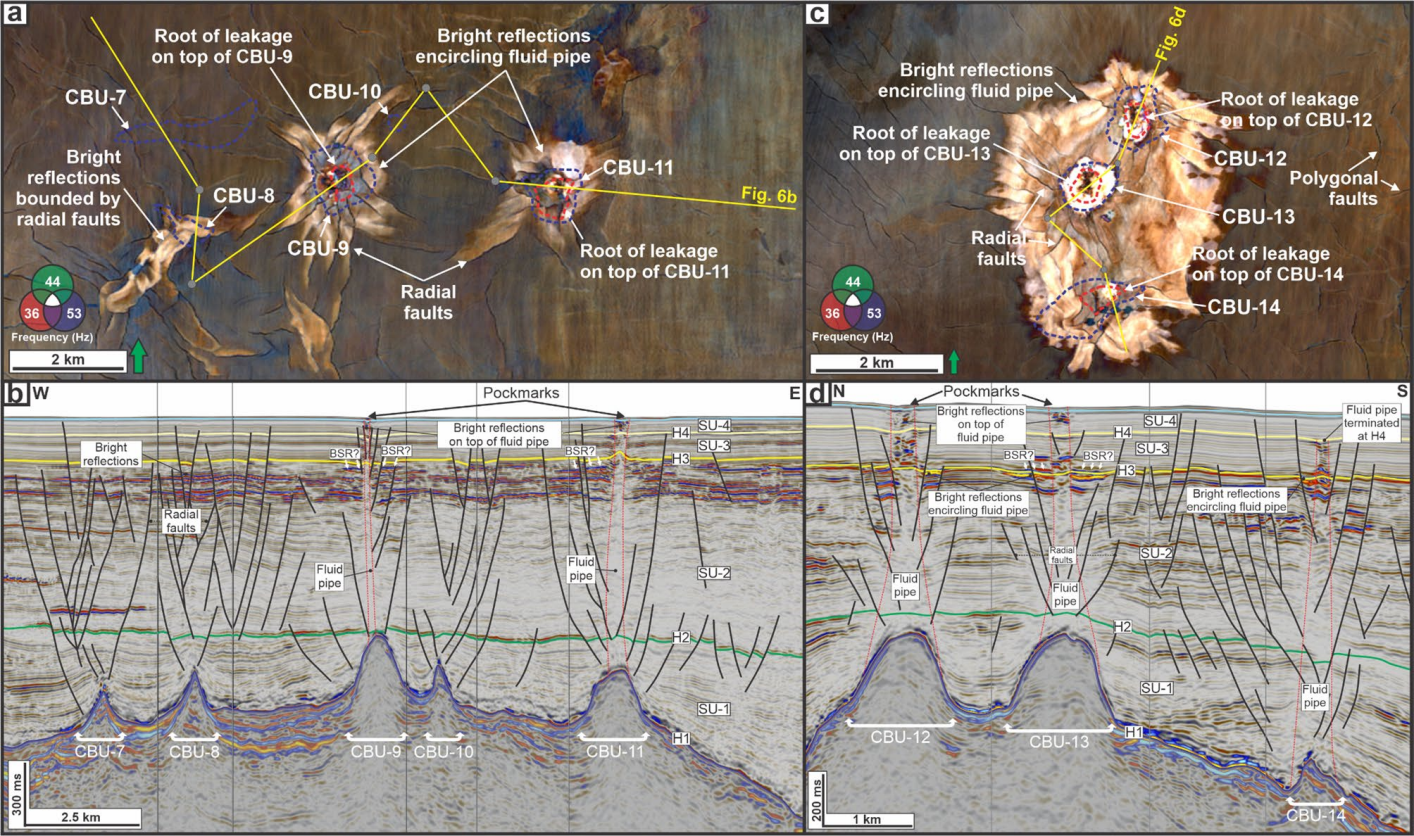
(**a**) LZ-3: RGB-blended spectral decomposition of H3, illustrating the arrangement of fluid pipes, radially outward-striking faults, surrounding polygonal faults, and bright reflections encircling the fluid pipes. (**b**) Interpreted seismic section across LZ-3. (**c**) LZ-4: RGB-blended spectral decomposition of H3, illustrating the arrangement of fluid pipes, radially outward-striking faults, surrounding polygonal faults, and bright reflections encircling the fluid pipes. (**d**) Interpreted seismic section across LZ-4.

LZ-3 comprises two pipes above build-up crests (CBU-9 and CBU-11) (Fig. 6a,b). The pipe above CBU-9 is 1323 m high and 633 m wide at its base, whereas the pipe above CBU-11 narrows upward and measures 1525 m in height with a root width of 985 m (Fig. 6b). The upper part of these pipes exhibits bright reflections and coincides with pockmarks on the seabed.

In the northern part of the study area (LZ-4), three pipes occur above build-up crests (CBU-12 to CBU-14) (Fig. 6c,d). Two pipes (CBU-12 and CBU-13) are comparatively short (899 m and 898 m high), with basal widths of 753 m and 843 m, respectively; the third (CBU-14) is taller (1266 m) and narrower (496 m at the base) and its terminus is defined by H4 (Fig. 6d).

In summary, all 14 CBUs are associated with a small-offset fault network (Figs. 5 and 6); however, only 8 of the 14 CBUs are associated with fluid pipes. At LZ-3, for example, five CBUs are present, but pipes occur only above two CBUs (i.e., CBU-9 and CBU-11; Fig. 6b). Of the eight pipe-associated CBUs, one is overlain by a pipe that terminates at H4 (i.e., CBU-14; Fig. 6d), whereas the remaining seven reach the seabed and are spatially coincident with pockmarks.

### Small-offset fault networks (polygonal and radial faults)

**Fig. 7:**
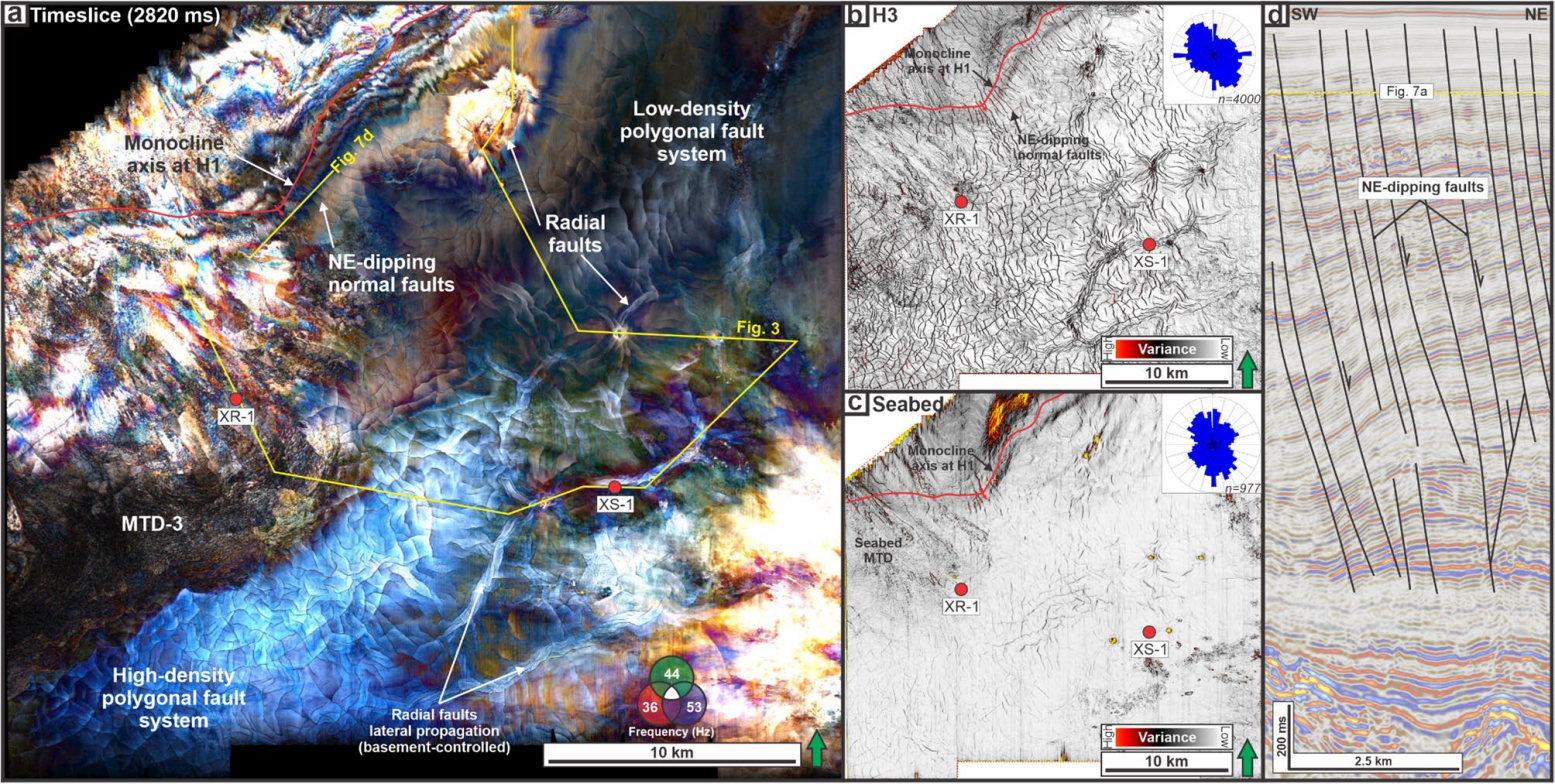
(**a**) Time slice at 2820 ms (see Fig. 3 for slice position) showing NE-dipping normal faults adjacent to a major fault zone in the northwest. Polygonal-fault lineaments dominate the central area, with higher lineament density in the southwest than the northeast. Radial lineaments in the southeast around LZ-2 and LZ-3 extends away from the related fluid pipes. Variance map at (**b**) horizon H3 and (**c**) seabed, showing the polygonal-fault lineament network. Inset rose diagram summarizes the azimuth distribution of fault-related lineaments extracted from the variance attribute. (**d**) Seismic section showing the NE-dipping normal faults.

In seismic section, numerous small-offset breaks in reflections continuity that extend from H1 toward the seabed are variably developed through the post-rift stratigraphy, with overall decreasing discontinuity density upward (Figs. 3 and 5, 6). These reflection discontinuities have polygonal planform geometry as depicted in a time slice of RGB-blended spectral decomposition (Fig. 7a). This polygonal network of reflection discontinuities is termed polygonal faults^15,56^. The highest polygonal faults density occurs in the southwestern part of the study area, with sparser distributions toward the northeast (Fig. 7a), broadly following the thickest post-rift corridor mapped between H1 and the seabed (Fig. 1d).

A second set of small-offset discontinuities is concentrated around the carbonate build-up crests and fluid pipes (particularly in the vicinity of LZ-2, LZ-3, and LZ-4; Figs. 5c-d and 6). In map view, these discontinuities show a radial planform, radiating outward from build-up crests and locally intersecting the polygonal network to form zones of structural interaction (Fig. 7a). Away from the fluid pipes (e.g., around LZ-2), some of these radially arranged discontinuities extend laterally beyond the immediate build-up crest region (Fig. 7a). These radially arranged sets are interpreted as radial faults, which are outward-radiating fault patterns around local structural highs^25,59^. In addition, adjacent to the major fault in the NW part of the study area (see Fig. 1c and e), a set of NE-dipping normal faults that trend 135°-315° with vertical displacements of up to c.60 ms are observed (Fig. 7d).

In seismic section, both polygonal and radial faults show an upward decrease in displacement, from c. 50 ms at H1 to negligible (i.e., not resolvable) displacement at H4 and/or the seabed (e.g., Figs. 5d and 6b, d). To summarise the overall orientation of the small-offset faults network, fault-related lineaments were extracted from variance attribute maps at H3 and the seabed. The extracted lineaments show a broad range of orientations at both horizons, with a clear dominant mode at 140°-320° (NW-SE) for H3 and a different dominant mode at 350°-170° (NNW-SSE to N-S) for the seabed.

### Bottom simulating reflections (BSRs) and other amplitude anomalies

**Fig. 8:**
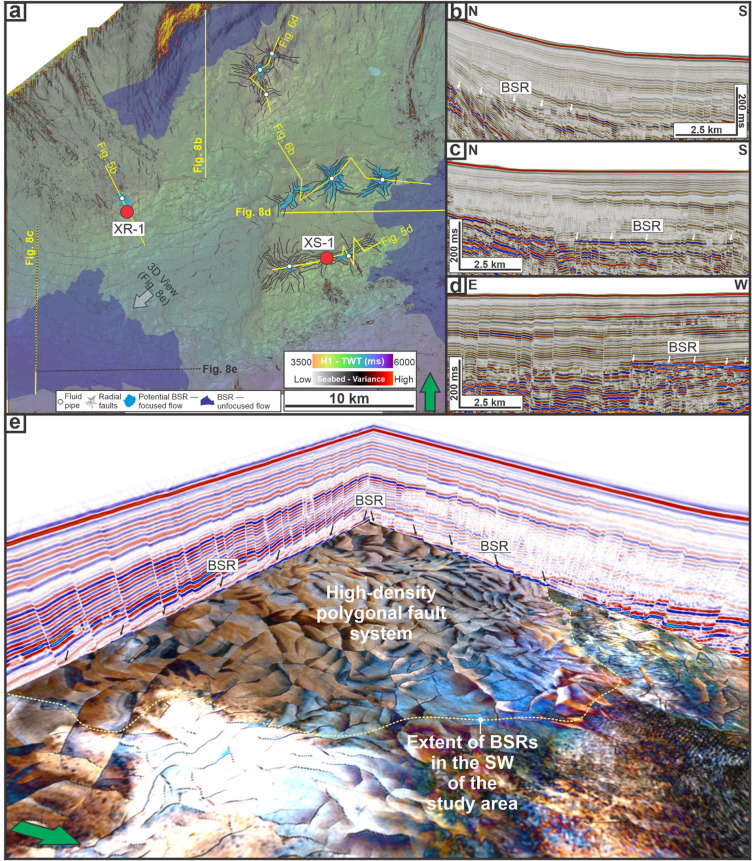
Gas hydrate distribution inferred from bottom-simulating reflections (BSRs). (**a**) Time structure map of H1 overlaid with a transparent seabed variance map. Two types of BSRs are identified: light blue indicates potential BSRs associated with focused fluid flow (i.e., fluid pipes), while dark blue highlights BSRs associated with unfocused, laterally extensive flow (e.g., polygonal faults). (**b-d**) Seismic sections showing BSRs associated with polygonal faults. (e) 3D perspective view of seismic sections and a time slice at the BSR level (see panel (a) for viewpoint), showing the close association between BSRs and high-density polygonal fault systems.

Laterally continuous, seabed-mimicking reflections with an opposite polarity relative to the seabed are observed in several parts of the study area (Fig. 8). These reflections cross-cut the surrounding stratigraphy and are commonly associated with high-amplitude reflections beneath. These reflections are observed in three main areas (Fig. 8a): (i) the northwest at a depth of 2074 m (Fig. 8b), (ii) the southwest at a depth of 2187 m (Fig. 8c), and (iii) the eastern area at a depth of 2169 m (Fig. 8d). Based on their seismic characteristics, these features are interpreted as bottom simulating reflections (BSRs)^60,61^. Spatially, the northwest BSR coincides with the NE-dipping normal faults adjacent to the H1 monocline (Figs. 7 and 8a-b), while the southwest and eastern BSRs occur within domains of polygonal faulting (Fig. 8a, c-d). In three-dimensional view, the coincidence between the southwest BSR with the high-density polygonal-fault zone is depicted (Fig. 8e).

In addition to the laterally continuous BSRs, laterally restricted, low-frequency, negative-polarity reflections occur at multiple locations adjacent to, and commonly encircling, fluid pipes (Figs. 5 and 6). These reflections are observed beneath MTD-2 at LZ-1 at 2193 m (Fig. 5a-b) and adjacent to fluid pipes associated with CBU-3 and CBU-4 (located above H3 at LZ-2 between 2111 and 2144 m; Fig. 5d), CBU-9 and CBU-11 (located below H3 at LZ-3 between 2160 and 2179 m; Fig. 6b), and CBU-13 (located at H3 within LZ-4 between 2111 and 2127 m; Fig. 6d). In some places, these reflections are relatively flat and do not show a clear cross-cutting relationship with the surrounding stratigraphy, for example above CBU-9 and CBU-11 (Fig. 6b) and CBU-13 (Fig. 6d). Accordingly, we interpret these reflections either as potential BSRs (albeit with lower confidence than the laterally continuous BSRs; see Fig. 8) or as localised free-gas accumulations associated with focused fluid flow through the pipes^20^.

Across the post-rift succession, lateral amplitude variability includes enhanced amplitude reflections (EARs) and dim-amplitude/wipe-out zones (Fig. 3a). EARs occur as laterally extensive, high-amplitude reflection events that are predominantly located in the upper part of SU-3 (Fig. 3b). In contrast, dim-amplitude/wipe-out zones occur as intervals of reduced reflection continuity and lower reflection amplitude, expressed as degraded and less coherent reflectivity relative to adjacent strata (Fig. 3a). These wipe-out/dim-amplitude zones, most notably on top of LZ-2 and LZ-3, are seismically distinct from the fluid pipes (Fig. 3b). In comparable hydrate systems, the association of EARs with underlying seismic attenuation/wipeout is widely interpreted as a gas-charged interval (free gas) beneath or adjacent to the hydrate stability zone^13,22,62^, where gas-related scattering and attenuation reduce seismic continuity while high-amplitude reflections are maintained over a limited interval.

## Discussion

### Plumbing system architecture and gas hydrate distribution

#### Spatial relationships of fluid flow features

**Fig. 9:**
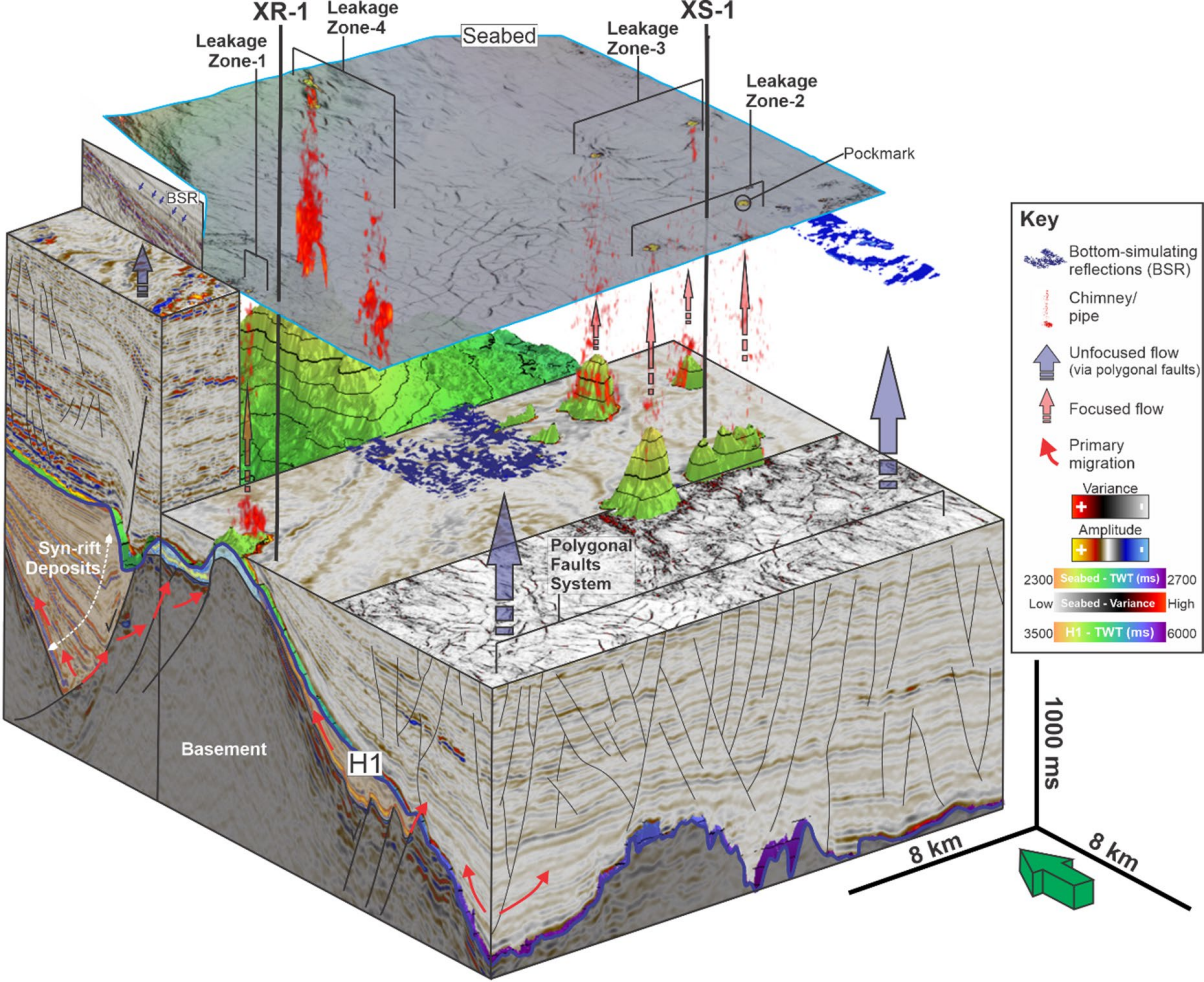
3D perspective view illustrating the architecture of the plumbing system in the study area.

The architecture of subsurface plumbing systems dictates the migration pathways of fluids from their sources to their final destinations, where they may accumulate or remigrate before ultimately being released at the seafloor^1^. In the study area, the mapped fluid flow features are consistent with two end-member plumbing systems: a focused flow system defined by vertically aligned leakage features, and an unfocused flow system defined by laterally extensive polygonal and radial fault networks (Fig. 9).

The focused flow system is defined by vertical alignment between deep reservoirs at H1, fluid pipes, and pockmarks on the seabed (Figs. 3 and 5 and 6). This configuration represents a secondary migration pathway originating from reservoir intervals, namely faulted carbonate platform blocks (LZ-1; Fig. 5a-b) and carbonate build-ups (LZs 2–4; Figs. 5c-d and 6). Similar associations between deep reservoirs and cross-stratal vertical conduits have been documented in other deep-marine settings, including offshore NW Australia^22^, the Barents Sea^63^, and offshore NW Borneo^20^.

The documented pockmarks are comparatively limited in number relative to the deep reservoirs on structural highs (Figs. 5 and 6). The spatial relationship between the reservoirs and the pockmarks suggests that seabed leakage is not an inevitable outcome above all reservoir highs. For instance: (i) CBU-2 that is penetrated by XS-1 contains a reported gas accumulation of 3 MBOE^34^(c. 18 BCF), yet no pipe is expressed above that crest (Fig. 5d), and (ii) the fluid pipe above CBU-14 terminates at H4 and thus is not expressed on the seabed (Fig. 6d). Therefore, charged reservoirs do not necessarily translate into seabed breach, instead factors such as the interplay between reservoir pressure and seal potential modulate the formation of fluid pipes and pockmarks^9,16^.

Evidences for active or geologically recent seepage are concentrated around this focused flow system. De Man, et al. ^36^ confirm the presence of gas hydrates across multiple locations within all four leakage zones (LZ-1 to LZ-4). At LZ-2 (Fig. 5c), piston coring was refused over a hardground, likely composed of methane-derived authigenic carbonates (MDACs) formed via microbial oxidation of methane at shallow depths^64^. Such lithification reflects localised, sustained fluid expulsion, reinforcing the interpretation of focused vertical leakage^58^. At LZ-4, chemosynthetic organisms were recovered from within the pockmarks (Fig. 6c-d), further indicating ongoing seepage^65^. The circular morphology of the pockmarks may suggest recent fluid discharge^18,66,67^, but would possibly be modified by strong bottom currents from the Indonesian Throughflow (ITF, see Fig. 1a).

The unfocused flow system is associated with fault networks that include: (i) widespread polygonal and radial fault networks (Figs. 8 and 9), and (ii) NE-dipping faults associated with the H1 monocline (Fig. 7a, d). These fault networks vertically coincide with laterally continuous BSRs, particularly where fault density is high (Fig. 8d). In addition, EARs and associated dim-/wipe-out amplitude zones are most prominent in the upper part of SU-2, where polygonal and radial faults are denser than in the underlying SU-1 (Fig. 3b). The presence of BSRs that indicate gas hydrate occurrence^61^, and EAR patterns that likely reflect gas-charged lithologies^9,20,22^, implies that the small-offset fault networks could facilitate upward fluid migration^3,9,21,68^.

The orientations of small-offset fault lineaments at H3 and the seabed show a broad azimuthal distribution (Fig. 7b, c), consistent with polygonal-fault populations that commonly lack a strong preferred strike due to their non-tectonic origin^69^. However, the dominant mode at H3 (140°-320°, NW-SE) and the different dominant mode at the seabed (350°-170°, NNW-SSE to N-S) suggest that additional factors influence the aggregate lineament pattern. Plausible contributors include (i) radial-fault trends around build-ups, and (ii) anisotropic tectonic forcing associated with adjacent regional structures (e.g., the Paternoster Fault Zone in the north and the WSFTB in the east; Fig. 1b). Together, these factors may contribute to the observed preferred modes in the small-offset discontinuity network and, in turn, may influence the connectivity of the faults.

Collectively, the spatial patterns of the mapped fluid flow features indicate that focused vertical conduits (pipes) and unfocused fault-related discontinuities coexist within the post-rift succession and may represent complementary parts of the plumbing architecture (Fig. 9). The former influences where leakage localises, while the latter influences where fluids are retained and redistributed^1,16,21^.

#### The role of mass-transport deposits (MTDs)

Mass-transport deposits (MTDs) are commonly expected to act as seals because shear-related compaction during their emplacement reduces porosity and pore-throat diameters, enhancing sealing capacity^38,70^. This compaction is typically expressed by elevated resistivity^71^, such as the higher resistivity values relative to surrounding strata of MTD-1 at XS-1 (Fig. 4). However, under elevated pore-pressure conditions, MTDs may become locally permeable^18^. For example, the presence of vertical fluid pipes that breach MTD intervals suggests that the internal heterogeneity and mechanical strength of these deposits might be insufficient to resist the pressure generated by underlying gas accumulations^16,18^. Most notably, the LZ-1 pipe exhibits an upward-converging geometry and the widest pipe root among the mapped fluid pipes, consistent with a broader, non-conical root geometry (Fig. 1c). Although imaging artifacts may contribute to apparent upward convergence, the lack of footprint-like lineations in map view supports a geological origin (e.g., Fig. 5a). In this context, the MTDs may have initially delayed vertical migration, enabling greater inferred overpressure buildup, thereby producing a broader conduit once failure occurred. Furthermore, the occurrence of potential BSRs and associated high-amplitude anomalies beneath the MTDs (Figs. 5b) suggests that, despite localised vertical leakage, MTDs can still retain gas away from the fluid pipe, promoting lateral gas migration beneath, or even within, their extent^72^. Therefore, MTDs have at least two roles: transient seals that can be breached under overpressure and leakage-modifying baffles that partition lateral versus vertical gas migration^18,72^.

### Evolution of focused fluid flow in the South Makassar basin

#### Styles of focused-flow expression

Three leakage styles within the focused fluid flow system in the study area are recognised. First, radial-fault patterns occur above several build-ups where vertical pipes are absent, often with bright amplitudes confined within fault-bounded zones (e.g., CBUs 2–3, 5–7, and 9; Figs. 5 and 6). These patterns radiate from build-up crests and locally overprint the broader polygonal-fault fabric, indicating build-up-centred deformation within the fine-grained seal. These radial fault networks may enable fluid migration and potentially precedes the development of vertical fluid pipes^9,25^, as evidenced by EARs in the upper part of SU-2 (Fig. 3b).

Second, arrested leakage is exemplified by the fluid pipe at CBU-14, which appears vertically continuous but terminates at H4 without forming a pockmark (Fig. 6d). This suggests that fluid migration was either temporarily halted or redirected within the overburden, possibly due to local seal integrity or insufficient overpressure to breach the seafloor. However, the continuity of the pipe below H4 may still indicate a presently active or intermittently reactivated pathway, where expulsion has yet to reach the surface^16,27^.

Third, breaching leakage is represented by vertically continuous fluid pipes that extend from the top carbonate reservoirs (H1) to the seabed, which could have either straight (e.g., CBU-9, Fig. 6b) or upward-converging geometries (e.g., at LZ-1 in Fig. 5b, CBU-1 in Fig. 5d, CBU-11 in Fig. 6b, and CBU 12–13 in Fig. 6d). The scale and geometries of these fluid pipes are comparable to other pipes documented in deep marine basins^16^. Together, these three styles define a spectrum from build-up–centred radial faulting to arrested and breaching focused conduits^11,16,73^.

#### Stages of development

**Fig. 10:**
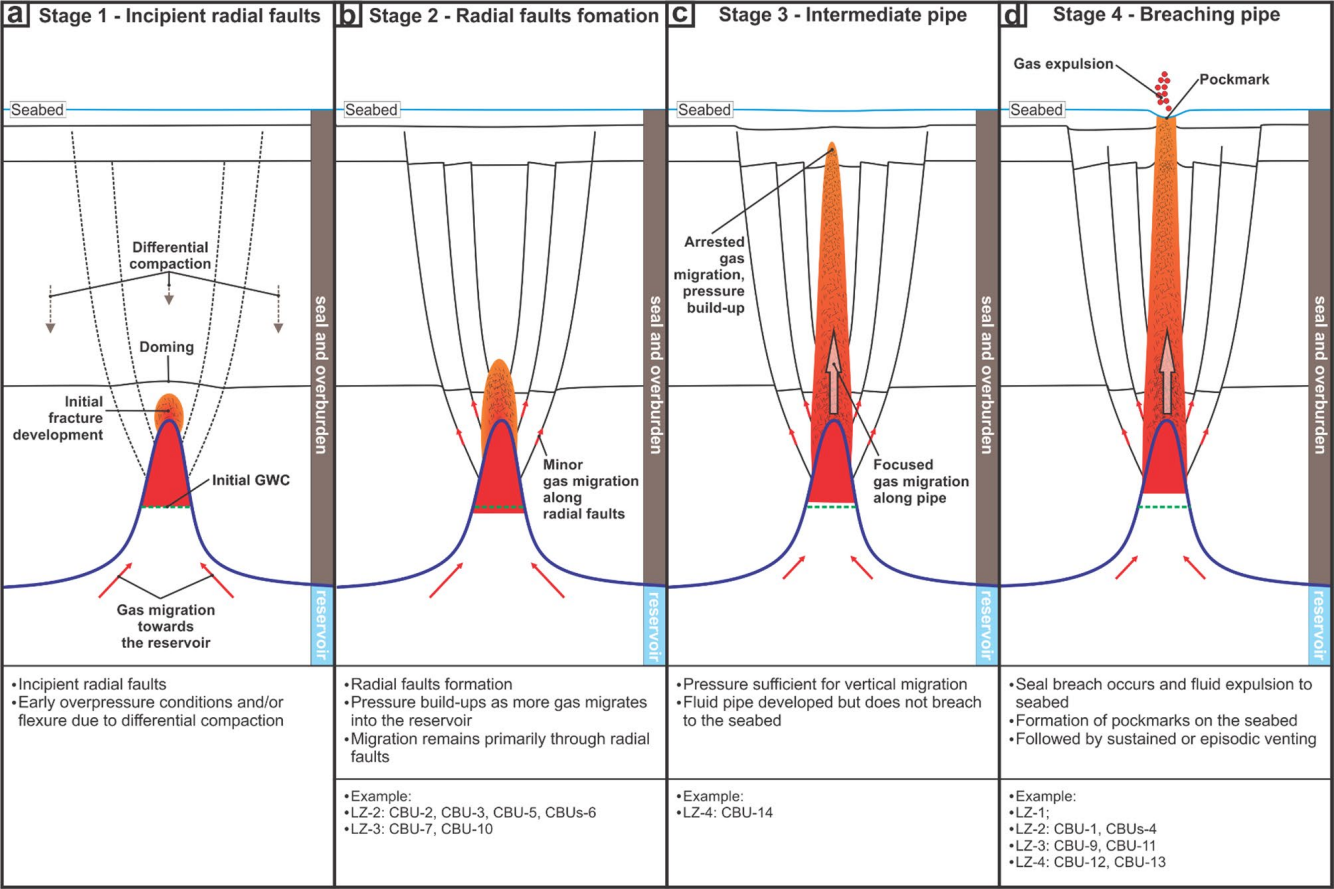
Schematic illustration of the stages of fluid pipe development, progressing from incipient radial faulting to the formation of mature, breaching fluid pipe and associated pockmarks.

A progressive evolution of focused leakage is inferred from the observed spectrum of focused-flow styles (Fig. 10). In the first stage, overpressure is inferred to begin accumulating at the top of carbonate reservoirs, where conical, high-relief geometries may impose localised stress perturbations in the overlying fine-grained seal and promote the formation of incipient radial fractures (Fig. 10a). In contrast, star-like radial patterns are not evident above non-conical reservoir geometries such as the low-relief carbonate platform at LZ-1 (Fig. 5b) and the elongated build-up geometry at CBU-7 (Fig. 6a), implying that reservoir geometry may serve as a key control on whether radial faulting develops.

Although clear doming is not apparent in present-day structure, the tendency for maximum radial-fault displacement to occur near build-up crests (at H1) suggests that fault initiation in the first stage may have been triggered by subtle, localised uplift (Fig. 10a). This interpretation comparable to observations from other settings, such as hydrothermal vents and sediment injection mounds in the Vøring Basin^74^and buried mud mounds in the Canterbury Basin^25^, where doming has been shown to induce radial extension within otherwise polygonally-faulted successions.

Alternatively, differential compaction of claystone-dominated seal and overburden draped over a stiff carbonate build-up can generate flexural curvature and crestal extension that promotes normal faulting^75,76^. This is linked to the greater cumulative compaction beneath thicker flank overburden than above the thinner crestal cover of the carbonate build-up (Fig. 10a). Over time, continued compaction can cause modest throw loss (typically < 20%) and preferentially reduce the shallow preserved throw^77^, such that the deeper throw maximum is more likely to be retained^77,78^. In practice, both localised uplift and differential compaction could operate together. Therefore, further studies are needed to investigate the relative roles of localised uplift versus differential compaction, including the interaction and formation timing of polygonal and radial faults^57,69^.

As pressure builds and deformation progresses, radial faults extend vertically toward the seabed with diminishing throw, forming a fully developed fault network. This marks the second stage of evolution, where radial faults act as a seal bypass system that facilitates upward fluid migration (Fig. 10b). In this stage, localised vertical flow may be distributed through the radial faults, but flow localisation continued to occur, enabling hydraulic fracturing to progress^16^.

In the third stage, continued hydraulic fracturing allowed fluid localisation (*sensu* Cartwright and Santamarina^16) ^to progress, forming pipe-like features identifiable in seismic as vertical columns of disrupted, transparent reflections^11^. However, leakage remains partially arrested: pressure is sufficient to localise flow but not yet overcome the capillary entry pressure of the full overburden. This behaviour is consistent with capillary invasion concepts in which buoyancy-driven gas ascent can be limited by capillary barriers and heterogeneity, constraining conduit width and vertical reach^80^. Thus, the observed pipes likely represent pre-breach conduits awaiting further pressure buildup to progress upward.

The final stage of the continuum is characterised by the formation of breaching fluid pipe, where sustained overpressure drives vertical conduits through the entire seal interval to the seafloor, resulting in focused venting and pockmarks formation (Fig. 10d). This interpretation suggests the breaching pipes as efficient vertical migration pathways^3,16^, with potential implications for hydrate formation and stability near the leakage features^20,79^.

#### Controls on stage development

Progression from build-up-centred deformation to a through-going, seabed-breaching conduit is interpreted to reflect the interplay between (i) the driving force for vertical expulsion (reservoir overpressure and its persistence) and (ii) the resistance imposed by the overburden (capillary entry thresholds, mechanical competence, and heterogeneity), with reservoir-top geometry modulating where deformation is preferentially initiated^11,16,80^. This is reflected by the fact that different styles of focused-flow expression can occur within the same leakage-zone cluster, where variations in reservoir-top geometry and local pressure conditions, together with local seal architecture, may modulate the balance between driving force and resistance. For instance, at LZ-4 (CBU-12 to CBU-14; see Fig. 6c-d), the interplay is expressed by contrasting outcomes over short lateral distances. CBU-12 and CBU-13 are associated with the shortest fluid pipes (c.900 m) and the deepest pockmarks (up to 18 m). Although pockmark depth and preservation may be influenced by post-formation modification^18^, this inverse relationship may indicate reduced vertical resistance along shorter travel paths, potentially favouring more abrupt venting (i.e., blowout pipe *sensu* Cartwright, et al.^3^). In contrast, the fluid pipe above CBU-14 terminates at H4 and is not expressed as a pockmark (Fig. 6d), indicating that upward propagation can stall where the available pressure drive does not exceed local capillary and/or mechanical thresholds^9,16,27^.

While time scale for each stage cannot be constrained by our seismic data, a hydro-mechanical modelling study suggests that transition from fracture localisation to pipe breakthrough (Stage 2–4) can occur rapidly, with pipe-propagation speed on the order of 30 cm/yr through mudstone-dominated seal^81^. Accordingly, further work is needed to better constrain stage durations in our study area, which would require independent age control and geomechanical analysis beyond the scope of this study.

### Implications for natural methane emissions and geological storage security

The architecture and evolution of fluid migration pathways in the South Makassar Basin, offshore Indonesia, provide key insights into the challenges for natural methane emissions and monitoring long-term subsurface storage. The basin hosts both focused and unfocused fluid flow plumbing systems, each with distinct implications for fluid containment and leakage risk.

In terms of natural methane emissions, polygonal fault networks enable slow methane release, while fluid pipes provide rapid venting pathways (Fig. 9). In addition, changes in ocean currents temperature could induce gas hydrate dissociation and thus increasing methane release to the ocean^82,83^. For instance, over the past 30 years, a 1°C warming of the West Spitsbergen Current has driven retreat of the gas hydrate stability zone and the appearance of > 250 methane plumes along the West Spitsbergen margin^83^. In contrast, the Indonesian Throughflow (ITF) has intensified in recent decades, increasing heat transport and warming in the Makassar Strait^84,85^, but has not yet been directly linked to hydrate destabilisation. Given extensive hydrate-bearing sediments in ITF-influenced areas, continued monitoring of ITF variability and subsurface temperatures is needed to evaluate future hydrate sensitivity and potential methane release^32^.

From a storage security perspective, neither system is low risk (Fig. 9). Polygonal and radial faults within the thick post-rift mudstones may facilitate slow fluid migration and act as semi-permeable baffles, as demonstrated in other fine-grained seals globally^3,9,21,57,68,86^. These features are often considered advantageous for containing injected fluids such as CO₂ due to their low vertical permeability^3^. However, in this study, the presence of BSRs directly above these faults might indicate that gas has migrated through them over geological time, revealing their potential long-term transmissivity. Focused vertical fluid flow features, including fluid pipes rooted at faulted carbonate build-ups, present even higher leakage risk. These structures can breach the sealing units and connect deep reservoirs to the seabed, as recently documented in the offshore NW Australia^9^, the South China Sea^87^, and the Gulf of Mexico^18^. The coexistence of both plumbing systems suggests that spontaneous pipe formation, mechanical seal failure, and structural focusing all contribute to seal bypass, reinforcing the need for high-resolution risk assessments prior to any subsurface storage application.

Notably, in the absence of injection, 8 of the 14 carbonate build-ups in our study area are associated with fluid pipes. This reflects long-term natural overpressure development and suggests spatial and temporal variability in leakage thresholds governed by local gas charge, pressure dissipation, and seal strength/stress state^3^. Superimposed on such baseline conditions, CO₂ injection could generate short-timescale pressure build-up and a pressure perturbation footprint that extends well beyond the CO₂ plume, with implications for brine displacement and geomechanical integrity^88–91^. As a screening consideration, injection-driven pressurisation is expected to be most critical for caprock integrity where lateral pressure dissipation is limited and the margin to fracture initiation or fault reactivation is small^90,92,93^. In settings with established seal-bypass architectures, the natural system provides evidence for episodic leakage in the geological past, and added injection-related pressure could possibly increase the likelihood of reactivating dormant pathways above the reservoirs that currently show no pipes^3,90,92,93^.

Quantifying the relative contributions of injection-driven and natural pressure build-up would require dedicated coupled flow-geomechanical modelling and is therefore beyond the scope of this study. Nevertheless, global assessments support the feasibility of safe geological storage under well-characterised and properly managed conditions; for example, Alcalde, et al. ^94^ estimate > 98% retention over 10,000 years for well-regulated storage scenarios. The key implication here is that natural plumbing architectures like those observed in this basin must be explicitly mapped, classified, and potentially excluded from storage target zones.

## Conclusions

This study provides an integrated analysis of subsurface plumbing architecture, focused-flow evolution, and associated implications for gas hydrate distribution, seal integrity, and long-term subsurface storage in the South Makassar Basin, offshore Indonesia. By utilising high-quality, post-stack time-migrated (PSTM) 3D seismic and well data, we identified two fluid flow systems in the basin. First, focused vertical pathways expressed as fluid pipes that link deep carbonate reservoirs to the shallow section and, in many cases, to seabed pockmarks. Second, unfocused, laterally extensive pathways expressed by polygonal and radial fault networks that coincide with widespread bottom-simulating reflections (BSRs) indicative of gas hydrate occurrence. Based on these observations we propose a four-stage continuum of focused flow development, beginning with radial fault initiation due to localised stress above carbonate build-ups, followed by pipe formation, and eventual breaching of the seal to form pockmarks on the seabed. These stages reveal a structurally mediated continuum of seal bypass development, controlled by reservoir geometry, overpressure buildup, and mechanical heterogeneity of the overburden. Our findings show that natural methane emissions may occur via both focused and unfocused pathways, and that their interaction with the Indonesian Throughflow (ITF) warming and gas-hydrate stability warrants further investigation. More importantly, our study highlights the importance of detailed architectural mapping and classification of fluid migration pathways when evaluating basin suitability for CO_2_ storage or other containment-based applications.

## Data Availability

The data utilised in this study were provided by Information and Data Centre, Ministry of Energy and Mineral Resources (PUSDATIN ESDM) of the Republic of Indonesia, which were used under licence for the current study and so are not publicly available. Data from PUSDATIN ESDM, however, are publicly available for request through datamigas.esdm.go.id.
